# Editorial: Genomic Characterization of Emerging Human Fungal Pathogens

**DOI:** 10.3389/fgene.2021.674765

**Published:** 2021-03-25

**Authors:** Bridget M. Barker, Christina A. Cuomo, Nelesh P. Govender

**Affiliations:** ^1^Pathogen and Microbiome Institute, Northern Arizona University, Flagstaff, AZ, United States; ^2^Broad Institute of MIT and Harvard, Cambridge, MA, United States; ^3^National Institute for Communicable Diseases, National Health Laboratory Service, Johannesburg, South Africa; ^4^School of Pathology, University of the Witwatersrand, Johannesburg, South Africa; ^5^Division of Medical Microbiology, University of Cape Town, Cape Town, South Africa

**Keywords:** Candida, black fungi, *Aspergillus*, Mucoralesi, *Coccidioides*, *Paracoccidioides*, RNA-seq

Human fungal pathogens pose a major clinical challenge with the emergence of new species, expanded geographic ranges impacting new populations, and the increasing frequency of antifungal drug resistance. Rapidly addressing these new threats can leverage genomic approaches to characterize new species or lineages and trace the origin and spread of outbreaks. Genomic data can also guide the improvement or development of diagnostic approaches to detect new species and genetic elements conferring drug resistance. This issue features reports of species from across the fungal kingdom, and highlights the range of genomic approaches being used to study these important pathogens.

Bombassaro et al. compared the enzyme-coding gene profile of *F. pugnacius* to other neutropic black fungi such as *Cladophialaphora bantiana, Exophiala dermatitidis*, and *Rhinocladiella mackenziei* and examined the virulence of this pathogen in invertebrate and mouse models of infection. *F. pugnacius* is a very rare human pathogen with a single strain described to date in a case of chromoblastomycosis with secondary neurotropic dissemination (phaeohyphomycosis) from a presumed subcutaneous source. Its sibling species, *F. pedrosoi* and *F. nubica*, cause chromoblastomycosis which is characterized by muriform cells in subcutaneous tissue usually of the extremities, while another related species*, F. monophora* causes primary brain infection. In this paper, the authors describe a 34.8 Mb genome and 12,217 protein-coding genes associated with extremotolerance, virulence and neurotropism. *F. pugnacius* survived in an invertebrate model with lower mortality than *F. monophora* and *F. pedrosoi* but with high colony-forming units, melanisation of larval hemolymph, and melanised hyphae in tissue. In a murine intradermal model, muriform cells were found in the footpads but with no dissemination; whereas with intraperitoneal inoculation, the pathogen was recovered from multiple tissues. The authors conclude that this is probably an opportunistic fungus with enhanced ability to tolerate stress and with adaptability to new niches.

Dos Santos et al. characterized and compared six clinical strains of *Aspergillus fumigatus*, a known major pathogen and cryptic species, *A. lentulus* (*n* = 5) and *A. fumigatiaffinis* (*n* = 4). Based on a *Galleria mellonella* model, they describe high heterogeneity in virulence across strains in each of the three species; in general, the cryptic species were as virulent as *A. fumigatus*. They performed susceptibility testing using the EUCAST method for polyenes, azoles, echinocandins, and allylamines. While *A. fumigatus* had lower amphotericin B minimum inhibitory concentrations, susceptibility in general could not be predicted by cryptic species-level identification. They found a paralog of the *CYP51A* gene in *A. fumigatiaffinis* similar to *CYP51C*. They found no *FKS* mutations but some changes in *FKS* hotspot region 2 specific to the cryptic species. Based on genomic sequencing, they identified single nucleotide polymorphisms, virulence genes, and performed a phylogenomic analysis. Most genes were shared among all three species, including most virulence genes.

Soare et al. reviewed biological and virulence determinants of fungi in the Order Mucorales. They describe that the Order Mucorales diverged from common ancestor with Ascomycetes and Basidiomycetes 800 million years ago. Next-generation sequencing has facilitated an understanding of the genomic architecture of fungi in this Order (including an ancestral whole genome duplication event in some genera), virulence (nearly 800 candidate genes are truncated, discontiguous, or absent in avirulent strains), host response and important host-pathogen interactions, resistance, diagnostics (no specific biomarkers are available; molecular tests are few and not validated though a pan-Mucorales COTH gene family not present in *Aspergillus* can be amplified using a single primer set) and novel therapies (mycoviruses).

Misas et al. discuss new mitochondrial sequence data of *Paracoccidioides brasiliensis* strain Pb18 and *P. americanum* strain Pb03. They confirm the observation of a previous incomplete assembly that the *NAD5* gene contains a large insertion between exons 2 and 3 by using long read sequencing and additional sequencing data for bioinformatic assembly and annotation approaches. The authors point to a lack of well-assembled mitochondrial genomes and a need to functionally understand the mitochondria in Onygenalean fungi in general.

Mead et al. present new RNA-seq data for *Coccidioides posadasii*. The comparison of a chitinase 2/3 deletion mutant that is unable to complete the parasitic life cycle to the wild type parent strain reveals new insight into genes that might be responsible for the survival and growth of the fungus in the host. The volatilome was assessed for the mutant, and revealed numerous changes compared to the wildtype. Scanning electron microscopy suggests incomplete nuclear division in the mutant in response to loss of internal cell wall structure during spherule maturation. Several putative virulence factors are identified in this pathogen that causes coccidioidomycosis (valley fever) across the Americas.

Genomic approaches have played a key role in studies of the emergence and drug resistance of *Candida auris*. Chybowska et al. review nine areas where genomic data has been used to draw insights into this emerging species. Gade et al. characterize isolates from the *Candida haemulonii* species complex, the closest relatives of *C. auris*, which also are commonly resistant to antifungal drugs and are increasing in incidence. This study of global isolates included primarily *C. haemnulonii* and *C. duobushaemulonii*, with smaller numbers of *C. pseudohaemulonii* and *C. vulturna*. Genetic relationships and candidate mutations associated with azole resistance are described using whole genome sequence data. This use of genomic epidemiology reveals the need for further monitoring of outbreaks in healthcare settings as well as surveillance sequencing to follow the frequency of mutations associated with drug resistance.

Rodriguez-Leguizamon et al. examine a set of 10 *Candida albicans* isolates collected in Colombia for which initial studies indicated some shared properties with *Candida africana*. Multi-locus sequence analysis of seven standard genes showed that the *C. albicans* isolates belong to three clusters and do not appear closely related to *C. africana* isolates. Isolates showed varying levels of virulence in a *Galleria* model, and only one displayed resistance to fluconazole.

Thomas et al. carry out a co-expression network analysis using data from a prior RNA-Seq timecourse of *C. albicans* interactions with epithelial and endothelial cells. Analysis of the resulting network-extracted ontology (NeXO) identified clusters of genes enriched for roles in pathogenesis. In phenotypic tests of genes in clusters of interest revealed an unusual colony morphology for *PEP8*, which the authors go on to show is involved in hyphal development. This and other examples illustrate how the NeXO may be used to predict gene functional classes.

Usher et al. transformed a *C. glabrata* ORF library into *S. cerevisiae* and screened for transformants that were resistant to oxidative stress. This identified 16 candidates including genes known to have a role in stress protection, endosomal soring, transcription or translation, and epigenetic modification. One novel candidate gene named *ORI1* is shown to be required for oxidative stress resistance in *C. glabrata* using deletion and overexpression studies, and this role is further supported by a synthetic lethality screen.

In summary, this collection of manuscripts highlights important contributions to the field of human fungal pathogen genomics, and suggests future directions for research to advance progress in these important but often understudied human pathogens.

## Author Contributions

All authors listed have made a substantial, direct and intellectual contribution to the work, and approved it for publication.

## Conflict of Interest

The authors declare that the research was conducted in the absence of any commercial or financial relationships that could be construed as a potential conflict of interest.

